# Exercise as an Aging Mimetic: A New Perspective on the Mechanisms Behind Exercise as Preventive Medicine Against Age-Related Chronic Disease

**DOI:** 10.3389/fphys.2022.866792

**Published:** 2022-08-15

**Authors:** Wesley K. Lefferts, Mary M. Davis, Rudy J. Valentine

**Affiliations:** Department of Kinesiology, Iowa State University, Ames, IA, United States

**Keywords:** preventive medicine, exercise physiology, physiological mechanisms, stress adaptation, aging

## Abstract

Age-related chronic diseases are among the most common causes of mortality and account for a majority of global disease burden. Preventative lifestyle behaviors, such as regular exercise, play a critical role in attenuating chronic disease burden. However, the exact mechanism behind exercise as a form of preventative medicine remains poorly defined. Interestingly, many of the physiological responses to exercise are comparable to aging. This paper explores an overarching hypothesis that exercise protects against aging/age-related chronic disease because the physiological stress of exercise mimics aging. Acute exercise transiently disrupts cardiovascular, musculoskeletal, and brain function and triggers a substantial inflammatory response in a manner that mimics aging/age-related chronic disease. Data indicate that select acute exercise responses may be similar in magnitude to changes seen with +10–50 years of aging. The initial insult of the age-mimicking effects of exercise induces beneficial adaptations that serve to attenuate disruption to successive “aging” stimuli (i.e., exercise). Ultimately, these exercise-induced adaptations reduce the subsequent physiological stress incurred from aging and protect against age-related chronic disease. To further examine this hypothesis, future work should more intricately describe the physiological signature of different types/intensities of acute exercise in order to better predict the subsequent adaptation and chronic disease prevention with exercise training in healthy and at-risk populations.

## Introduction

Age-related chronic diseases (e.g., cardiovascular disease, chronic kidney disease, Alzheimer’s Dementia, Type II Diabetes, etc.) are among the most common causes of mortality and account for a majority of global disease burden (Yach et al., 2004; Kennedy et al., 2014; Murphy et al., 2018). Preventive lifestyle strategies such as exercise have emerged as potent, cost-effective means of reducing chronic disease risk (Sallis, 2009; Sepanlou et al., 2011; Bauer et al., 2014). Exercise has a critical role in disease prevention (Booth et al., 2012; Pedersen and Saltin, 2015; Sallis, 2015; Bennie et al., 2020) and has been proposed by the American College of Sports Medicine as a form of “medicine” (Church and Blair, 2009; Sallis, 2009, 2015). The protective effects of exercise on chronic disease risk are ultimately accumulated over time through physiological adaptations to the stress of exercise.

Acute exercise causes widespread physiological disruptions that require a complex, integrated response from the major physiological systems (autonomic, cardiovascular, metabolic, musculoskeletal, etc.) to meet the substantial requirements of human locomotion (Hawley et al., 2014, 2018). Repeated exposure to the physiological disruptions incurred by acute exercise (through exercise training) stimulate physiological adaptations that act to attenuate stress during subsequent exercise bouts (Małkiewicz et al., 2019). These exercise adaptations provide the foundation through which individuals can adapt and improve their ability to perform physical work (e.g., increase muscular power, endurance, aerobic capacity, etc.) and also prevent development of age-related chronic disease (Hawley et al., 2014, 2018). Thus, physiologic adaptations to exercise are the latent mechanisms through which exercise acts as medicine and reduces chronic disease risk. Despite seminal work that has identified several key mechanisms underlying the protective effects of exercise, there has yet to be an *overarching hypothesis* that explains broadly why or how it is that exercise protects against age-related chronic disease. We posit that exercise prevents age-related chronic disease because it acutely elicits physiological responses that mimic physiological changes seen with aging, the greatest contributing risk factor to all chronic disease (Bauer et al., 2014; Kennedy et al., 2014). Thus, we propose the hypothesis that exercise is “medicine” that protects against age-related chronic diseases because exercise can effectively simulate “aging.” This paper is not intended to comprehensively review the physiological adaptations to exercise or their specific benefits on health/disease (see prior reviews; (Hawley et al., 2014, 2018; Green et al., 2017; Tanaka, 2019; McGee and Hargreaves, 2020), rather, we will examine this hypothesis by comparing age-related physiological changes with those induced during acute exercise and integrate these responses within the context and implications of stress-induced adaptation. This is not a systematic review, rather, we conducted a literature search of original data and reviews (when appropriate) examining the physiological effects of acute exercise on the brain (cognitive, brain-blood-barrier), cardiovascular, neuroendocrine, inflammation/oxidative stress, metabolic, and musculoskeletal systems and then aligned those observations with literature describing changes seen with aging and age-related chronic disease.

## Effects of Aging and Exercise on the Brain

Aging is accompanied by natural reductions across multiple domains of cognitive function (memory, reasoning abilities, executive function, and processing speed) (Carlson et al., 1995; Hayden and Welsh-Bohmer, 2012; Salthouse, 2012; Harada et al., 2013). Increasing age is also associated with inflammation and oxidative stress that damages the cerebral microvasculature and decreases blood-brain-barrier integrity (Verheggen et al., 2020). Ultimately, reductions in higher-order cognitive processing (memory/executive function) and blood-brain-barrier permeability are implicated in the underlying pathology and presentation of dementia and Alzheimer’s disease (Salthouse, 2012; Harada et al., 2013; Gamba et al., 2015; Kirova et al., 2015).

Acute exercise imposes substantial stress on brain function and blood-brain-barrier integrity that parallel changes observed with age and cognitive disease. Acute exercise (particularly high intensity exercise) can impair higher order cognitive processing (e.g., executive function) through reallocation of mental resources (Audiffren et al., 2009) in an exercise intensity-dependent fashion (Lambourne and Tomporowski, 2010; Wohlwend et al., 2017). Exercise also acutely disrupts blood-brain-barrier integrity, with increased blood-brain-barrier permeability immediately following intense exercise (Sharma et al., 1991; Roh et al., 2017). This acute disruption in blood-brain-barrier integrity may be related to the effects of exercise on 1) oxidative-nitrosative stress (the origins of which are discussed further in subsequent sections) at the blood-brain-barrier interface that damages cells, reorganizes cytoskeletons, and increases inflammation (Sharma et al., 1991; Roh et al., 2017), 2) vasoactive effects of serotonin (Sharma et al., 1991), and 3) changes in cerebral blood flow patterns during exercise (e.g. increased pulsatile hemodynamics) (Armentano et al., 1991; Ogoh et al., 2005; Alwatban et al., 2020) which are linked with blood-brain-barrier damage and disruption (Jufri et al., 2015; Garcia-Polite et al., 2017; de Montgolfier et al., 2019).

## Effects of Aging and Exercise on the Cardiovascular System

Aging is associated with an increase in mean blood pressure, resulting from a steady rise in systolic blood pressure and a slight decline in diastolic blood pressure (Franklin et al., 1997). Age-related increases in blood pressure may stem from, and simultaneously promote, large artery stiffening (Henskens et al., 2008; Najjar et al., 2008; Kaess et al., 2012; Mitchell, 2014; Tarumi et al., 2014; Zhou et al., 2018), which amplifies the magnitude of forward traveling energy waves and increases pulsatile blood pressure and flow (Mitchell, 2014; Tarumi et al., 2014; Lefferts et al., 2020). Age-related increases in large artery stiffness may be due, in part, to endothelial dysfunction wrought by oxidative stress and subsequent reductions in nitric oxide bioavailability (Donato et al., 2015; LaRocca et al., 2017). Ultimately, age-related vascular dysfunction increases cardiac work (i.e., afterload) and results in left ventricular hypertrophic remodeling (Lovic et al., 2017; Yildiz et al., 2020) and diastolic dysfunction (Strait and Lakatta, 2012; Abdellatif et al., 2018). Cumulatively, age-related vascular and cardiac dysfunction are intrinsically linked with the risk and development of cardiovascular disease (Lakatta and Levy, 2003; Abdellatif et al., 2018).

The cardiovascular response during acute exercise is markedly similar to the detrimental, chronic changes in cardiovascular function seen with aging. Exercise produces a substantial blood pressure response [systolic pressures >190 mmHg in young adults (Sabbahi et al., 2018)] and increase in heart rate that stiffens the large arteries (Armentano et al., 1991; Studinger et al., 2003; Townsend et al., 2015). Increases in large artery stiffness during exercise (Studinger et al., 2003; Sharman et al., 2005; Pomella et al., 2018) are accompanied by increased forward wave energy (Jiang et al., 1995; Heckmann et al., 2000; Stock et al., 2021) and decreased wave reflection (Stock et al., 2021), ultimately contributing to greater pulsatile hemodynamics (Armentano et al., 1991; Ogoh et al., 2005; Alwatban et al., 2020). Additionally, exercise-induced acute increases in blood pressure may transiently impair endothelial function through a combination of mechanical distension/dilation of the artery, reductions in nitric oxide bioavailability, and endothelin-1 release during exercise (Millgård and Lind, 1998; Jurva et al., 2006; Gonzales et al., 2011; Morishima et al., 2020). This acute vascular response during exercise is further accompanied by a substantial (2–5-fold) increase in cardiac work (Channer and Jones, 1989; Rowland et al., 2002; Vega et al., 2017) that over time can stimulate ventricular remodeling in a similar manner to aging.

## Effects of Aging and Exercise on Neuroendocrine System

Aging impacts various neuro/endocrine regulatory systems throughout the body. Serum cortisol increases 20–50% throughout the adult lifespan (Chahal and Drake, 2007; Feller et al., 2014) owing to hormonal changes in the hypothalamic-pituitary-adrenal axis (Corazza et al., 2014). Aging is also associated with autonomic nervous system dysfunction manifesting as increased sympathetic and decreased parasympathetic nervous system activity (Pfeifer et al., 1983; Jandackova et al., 2016). Higher cortisol levels over time are associated with increased cardiometabolic disease risk and may compromise immune function in older adults (Corazza et al., 2014; Feller et al., 2014), whereas shifts in autonomic balance favoring sympathetic activity is an independent risk factor for cardiovascular disease (de Jonge et al., 2010; de Lucia et al., 2018). Both of these neuroendocrine responses to aging are mimicked by acute exercise. Cortisol levels increase during acute exercise in an intensity-dependent manner (Brandenberger and Follenius, 1975; Kanaley et al., 2001). Similarly, sympathetic nerve activity increases in exercising muscle and cardiac autonomic balance shifts to favor sympathetic over parasympathetic activity (Rowell, 1997; Michael et al., 2017).

## Effects of Aging and Exercise on Inflammation and Oxidative Stress

There is a well-established relationship between age and chronic low-level systemic inflammation (Ferrucci et al., 2010; Liberale et al., 2020). Circulating inflammatory markers increase with age in-part owing to increased chronic activation of the immune system (Liberale et al., 2020). Chronic inflammation with aging increases production of reactive oxygen (ROS)/nitrogen species (RNS) (Sergiev et al., 2015; Davalli et al., 2016). Higher levels of ROS/RNS promote cellular oxidative damage (cell membrane breakdown, protein modification, DNA damage) (Davalli et al., 2016) which can be further exaggerated by additional oxidative stress independent of ROS/RNS (Kudryavtseva et al., 2016). Ultimately, elevated markers of oxidative stress and systemic inflammation are strongly associated with increased risk of neurodegenerative, cardiovascular, and kidney disease, cancer, and dementia (Verbon et al., 2012; Marseglia et al., 2014; Kudryavtseva et al., 2016; Coen et al., 2018; Ferrucci and Fabbri, 2018; Senoner and Dichtl, 2019; Liberale et al., 2020).

Circulating inflammatory markers and oxidative stress also increase with acute exercise (Peake et al., 2005; Tsao et al., 2021). Acute exercise has been shown to increase pro-inflammatory cytokines such as interleukin (IL)-6 (Fischer, 2006), IL-7 (Małkiewicz et al., 2019), IL-10, C-reactive protein, and tumor necrosis-factor alpha (TNF-α) (Bernecker et al., 2013; Cerqueira et al., 2019; Fonseca et al., 2021) and initiate an inflammatory cascade (Powers and Jackson, 2008; McDonagh et al., 2014; Luca and Luca, 2019; Powers et al., 2020; Aragón-Vela et al., 2021). Additionally, exercise increases skeletal muscle ROS/RNS production *via* 1) electron leakage during oxidative phosphorylation within the mitochondria and NAD(P)H oxidase, 2) nitric oxide synthase activity within the skeletal muscle, 3) catecholamine and prostanoid release, and 4) ischemia/reperfusion-induced changes in xanthine oxidase activity, which ultimately contributes to oxidative stress, and subsequent cellular damage (Fisher-Wellman and Bloomer, 2009; McDonagh et al., 2014; Bouzid et al., 2015; Davalli et al., 2016; Powers et al., 2016; Petriz et al., 2017). As such, acute exercise can act as a pro-inflammatory stimulus that increases oxidative stress and damage in a manner similar to aging.

## Effects of Aging and Exercise on Metabolism

Advancing age is accompanied by alterations in both the metabolic pathways of energy production and mitochondrial function. Aging results in a steady rise in blood glucose concentration (Ko et al., 2006), driven in part by insulin resistance, exaggerated hepatic glucose production, and increasing cortisol levels (Satrústegui et al., 1986; Magnusson et al., 1992; Ko et al., 2006; Rizza, 2010). Similarly, aging and insulin resistance promote unrestrained lipolysis, which could contribute to systemic inflammation by increasing circulating free fatty acids (Reaven et al., 1989; Wende et al., 2012). Mitochondrial function is also impaired with aging, resulting in 1) increased sensitivity to ROS, 2) impaired oxidative metabolism, and 3) compromised mitochondrial membrane integrity (Shigenaga et al., 1994; Balaban et al., 2005). Mitochondrial dysfunction increases generation of oxidative byproducts (e.g. ROS/RNS) within the electron transport chain (Bratic and Larsson, 2013; Quinlan et al., 2013; Davalli et al., 2016) and thus accelerates age-related cellular damage (Genova and Lenaz, 2015). Taken together, these aspects of metabolic and mitochondrial dysfunction are associated with obesity, type II diabetes mellitus, obesity, fatty liver disease, cancer, sarcopenia and Alzheimer’s disease (Kim et al., 2001; Wende et al., 2012; Girousse et al., 2018; Yoo et al., 2019; Spitler and Davies, 2020; Paliwal et al., 2021).

Acute exercise also perturbs metabolic pathways, increases mitochondrial ROS production, and alters mitochondrial membrane permeability (Tonkonogi and Sahlin, 2002; Powers and Jackson, 2008). Acute exercise stimulates adipose tissue lipolysis, with low/moderate exercise eliciting a 2 to 5-fold increase in circulating free fatty acids for use in substrate metabolism (Havel et al., 1963; Ahlborg et al., 1974; Wolfe et al., 1990; Romijn et al., 1993; Ranallo and Rhodes, 1998; Burguera et al., 2000). Moreover, acute exercise also increases liver gluconeogenesis and hepatic glucose output *via* catecholamine release and sympathetic activity (Dibe et al., 2020) which aligns with changes in glucose production with aging.

## Effects of Aging and Exercise on the Musculoskeletal System

Skeletal integrity begins to decrease around 35–40 years of age, with postmenopausal women experiencing bone loss at a rate of approximately 1–2% annually (Riggs et al., 2008; Curtis et al., 2015) owing to disproportionate increases in bone breakdown versus buildup. Similarly, aging is also often accompanied by 1) muscle atrophy from imbalances between muscle protein synthesis and degradation in response to anabolic stimuli (Reynolds et al., 2002; Koopman and van Loon, 2009; Wall et al., 2015) and 2) reduced muscular force production (Zizzo, 2021). Age-related shifts in protein synthesis/degradation and reductions in force production may stem from free radical accumulation/oxidative stress and inflammation that activate proteolytic pathways, damage the muscle, and impair mitochondrial function (Guo et al., 2013; McDonagh et al., 2014; Fernando et al., 2019; Zizzo, 2021). Taken together, these musculoskeletal changes contribute to dyna-/sarco-penia and osteoporosis which have a profound impact on health and longevity with aging (Tagliaferri et al., 2015; Prawiradilaga et al., 2020).

Though exercise has long been known to stimulate bone mineralization and promote increased bone density, the initial response following any mechanical stimulus such as exercise is the resorption/breakdown of bone (Feng and McDonald, 2011). Similarly, exercise may acutely suppress muscle protein synthesis and increase protein degradation (Tipton and Wolfe, 1998; Kumar et al., 2009). Muscle force production also decreases following a bout of acute exercise (Howatson and van Someren, 2008) owing to, 1) inflammatory damage *via* increased mitochondrial reactive oxygen/nitrogen species within the working muscle (Powers and Jackson, 2008), and 2) structural damage (i.e., filament disintegration/misalignment, z-band streaming, excitation-coupling failure) incurred within the straining muscle (Fridén and Lieber, 1992). These effects of acute exercise ultimately contribute to initial reductions in voluntary force production following exercise (Howatson and van Someren, 2008).

## Implications of Adaptations to Exercise as an “Aging Stimulus”

**FIGURE 1:**
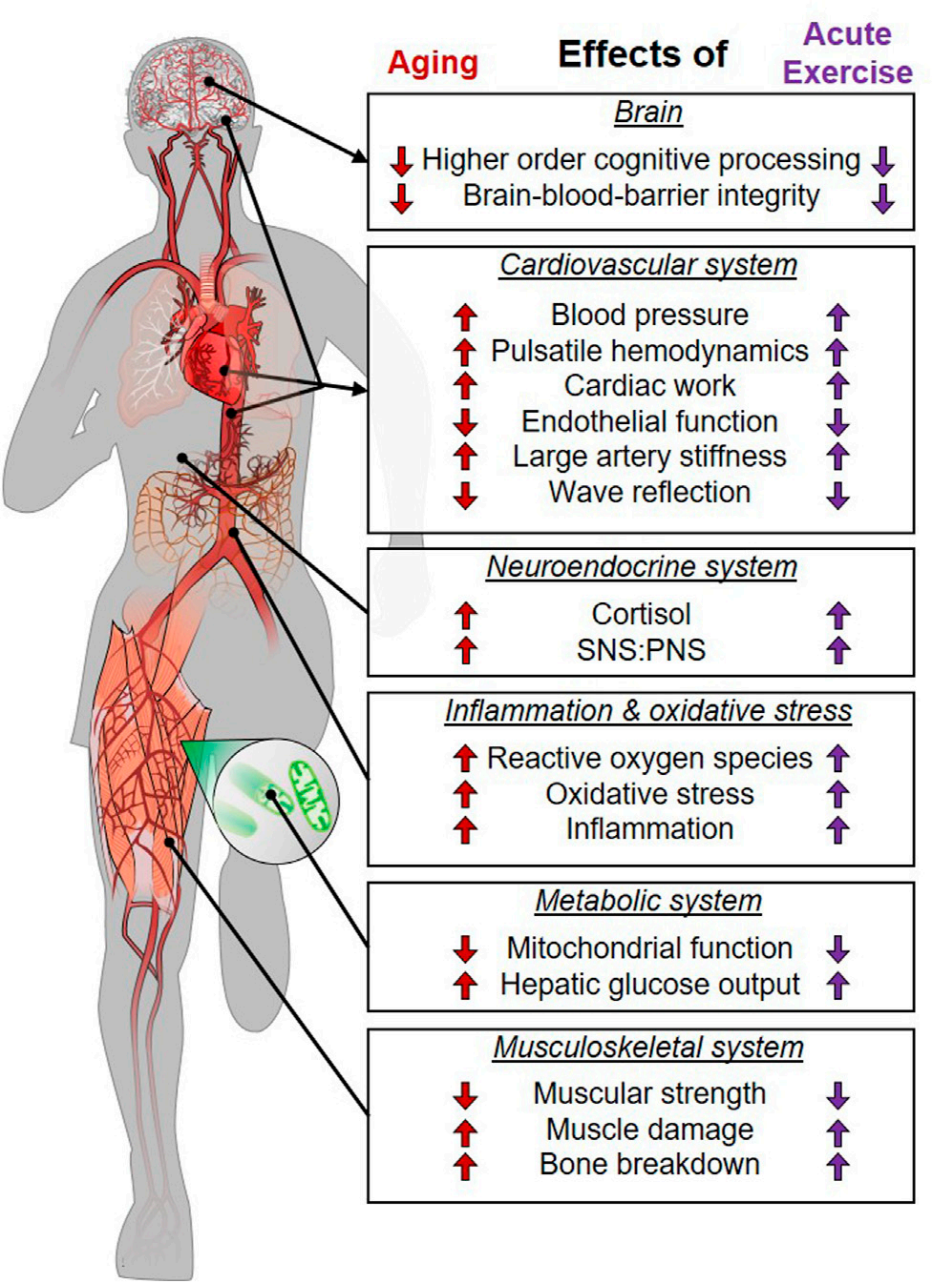
Parallels between the stress of aging/chronic disease and acute physical exercise. SNS, sympathetic nervous system; PNS, parasympathetic nervous system.

**FIGURE 2:**
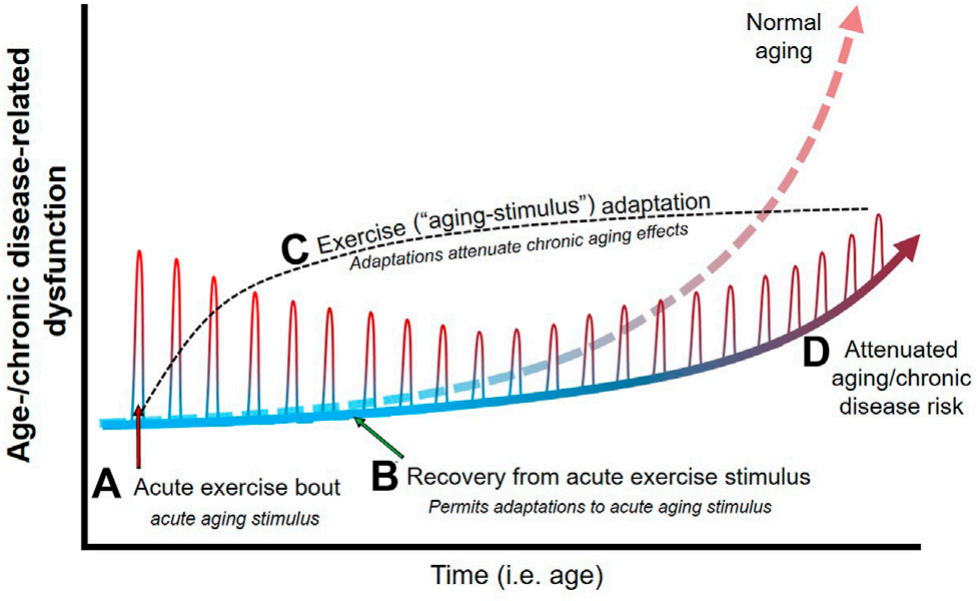
Theoretical effects of physical exercise as an aging stimulus on age-/chronic disease-related physiological dysfunction. Age-related dysfunction (e.g., cardiovascular, metabolic, muscular) generally increases steeply around middle-age into older age, and results in an increase in chronic disease risk. An acute bout of exercise **(A)** acts as an aging stimulus and elicits responses during exercise that mimic that of age-related dysfunction (e.g., increased large artery stiffness, inflammation, etc.). Cessation of exercise (i.e., removal of the acute aging stimulus) and proper recovery between exercise bouts/stimuli **(B)** permits adaptations **(C)** that serve to reduce the physiological stress during successive exercise (i.e., aging) bouts. Since acute exercise elicits physiological responses that parallel aging, exercise adaptations essentially prepare the body to endure less physiological stress and dysfunction when exposed to the effects of aging over time. As such, regular exposure to transient aging stimuli (i.e., regular physical exercise) elicits physiological adaptations that attenuate age-/chronic disease-related dysfunction **(D)**, and thus attenuates many of the detrimental physiological effects of age and protects against chronic disease development.

As outlined above, there is substantial evidence that the acute physiological response to exercise mimics physiological responses that occur with aging and age-related chronic disease (Figure 1). As such, acute exercise could be conceptualized as a transient bout of “aging.” The body naturally adapts to any stress (such as exercise) that disrupts homeostasis (Figure 2A). Proper adaptation to transient stimuli reduces stress during subsequent stressors (e.g., the next bout of exercise; see diminishing size of exercise-induced dysfunction in Figures 2A,B). For example, 1) exercise-induced increases in inflammation are attenuated following exercise training (Orlander et al., 1977; Fonseca et al., 2021), 2) increases in cardiac work are attenuated (e.g., lower heart rate) at a given workload following exercise training (Orlander et al., 1977), and 3) exercise training enhances antioxidant defense against exercise-induced oxidative stress (Bouzid et al., 2015). Parallels between the physiological stress of acute exercise and age-related chronic disease support the notion that repeated exposure to an exercise stimulus and the subsequent adaptations would protect against the physiological stress of aging and age-related chronic disease (Figure 2B).

If exercise is viewed as an aging mimetic, then more intense exercise should elicit a larger “aging” stressor and subsequent adaptation and protection against age-related chronic disease. Indeed, observational data suggest a dose-response relationship between exercise and physiological/health benefits, such that larger doses of exercise generally elicit greater protection (Ekelund et al., 2019; Shi et al., 2020; Aune et al., 2021). The protection afforded by exercise and the stress-adaptation cycle are maximized when the stress is transient and adequate recovery is allowed for adaptation (Figure 2C) (Booth and Laye, 2009). In the case of exercise, some data indicate extreme exercise volumes (e.g., marathons, ultramarathons) may be accompanied by pathological changes and a loss of health benefits, although this remains an area of debate (Eijsvogels et al., 2018; O’Keefe et al., 2020). Indeed, the line between physiological and pathological adaptations become blurred with high volumes of exercise being linked with risk of arrhythmias (Claessen et al., 2011; Andersen et al., 2013), cardiac dysfunction (O’Keefe et al., 2012; Rajanayagam and Alsabri, 2021), and myocardial injury (Neilan et al., 2006). Our hypothesis links to these observations since exposure to 1) extreme aging stimuli or 2) too frequent of exposure to an aging stimulus (preventing adequate recovery and adaptation) could contribute to negative (i.e., pathological) adaptations, accelerate physiological “aging,” and attenuate health benefits (Eijsvogels et al., 2018; O’Keefe et al., 2020). As such, the notion that exercise mimics aging provides insight into how exercise can both protect against age-related chronic disease and potentially give way to pathological changes under extreme exercise volumes.

## Alternative Perspectives and Limitations

We openly acknowledge that the actual cellular/molecular mechanisms driving acute and training responses to exercise may differ from those contributing to physiological changes with aging/age-related chronic disease (e.g., exercise and the cardiovascular demands required to meet metabolic output for musculoskeletal movement are fundamentally different mechanisms than those governing increases in blood pressure with aging such as degradation of elastin, microvascular rarefaction, endothelial dysfunction etc.). Many examples demonstrate the phenomenon of cross-tolerance, in which, despite diverse mechanisms, one stressor [e.g., exercise, environment (heat stress)] can confer protective benefits across other *different* stressors (Bond et al., 1999; Heled et al., 2012; Corbett et al., 2014; White et al., 2014; Wang et al., 2021). Consistent with this concept, our hypothesis is that the stimulus (e.g., an increase in blood pressure) for adaptation is similar between acute exercise and aging/age-related chronic disease and thus exercise adaptations may be mutually beneficial for both reducing the stress of subsequent exercise stimuli and aging/chronic disease pathways that involve that particular signal (e.g., blood pressure and cardio-/cerebro-vascular/cognitive disease).

Data indicate that lower intensity exercise/physical activity (e.g., walking) can confer mortality benefits in the absence of detectable physiological adaptations (Wasfy and Baggish, 2016). This raises the possibility that acute low intensity exercise 1) offers protection without adequately disrupting homeostasis and subsequent physiologic adaptations (contrary to our hypothesis), or 2) benefits age-related chronic disease burden through accumulation of diffuse, modest physiological adaptations that reflect a more modest exercise stimulus. Indeed, activities of daily living often viewed as “low” intensity (e.g., walking) are actually considered moderate intensity among older/deconditions populations (Sundquist et al., 2004; McPhee et al., 2016) and result in modest increases in energy expenditure (Maciejczyk et al., 2016), ventilation (Fusi et al., 2005), and cardiovascular stress (Renzi et al., 2010; Sugawara et al., 2015; Carter et al., 2018). Thus, even low-intensity exercise/physical activity may elicit similar directional physiological changes as “aging” and moderate-to-vigorous intensity exercise (as discussed above), albeit of smaller magnitude. This supports the idea that lower intensity activity patterns may need to be continued for longer periods of time to accumulate physiological benefits and reduce chronic disease risk (Carnethon, 2009). In the context of our hypothesis low-intensity exercise/physical activity likely elicits a smaller homeostatic disruption that represents a smaller “aging” stimulus, and thus more modest adaptations and benefits (in line with the dose-response literature). It is not surprising to see more sizeable benefits wrought from moderate and vigorous exercise intensities since these intensities can acutely elicit physiological responses comparable in magnitude to +10–50 years of aging (see Table 1) (Franklin et al., 1997; Hilbert et al., 2003; Ogoh et al., 2005; Ferrucci et al., 2012; Keith et al., 2013; Alwatban et al., 2020; Lefferts et al., 2020), and that exercise-trained older adults can be phenotypically similar to adults 40 years younger (Bhella et al., 2014; Fragala et al., 2019).

**TABLE 1:** Comparison of magnitude of acute exercise response with observed changes in the context of aging from select available literature.

Variable	Type of exercise	Acute exercise response	Aging	References
Cerebral pulsatility (MCA PI)	Moderate AE	+0.30au	+0.08/10 years from 45–85 years (totaling +0.30au across 40 years)^a^	Alwatban et al. (2020)
				Lefferts et al. (2020)
Pulse pressure	Mild AE	+10 mmHg	+22 mmHg^b^ from 30–84 years	Ogoh et al. (2005)
	Moderate AE	+24 mmHg	+35 mmHg^c^ from 30–84 years	Keith et al. (2013)
	Heavy AE	+37 mmHg		Franklin et al. (1997)
	Light AE	+21 mmHg		
Mean arterial pressure	Moderate AE	+7 mmHg	+7 mmHg^b^ from 30–64 years	Ogoh et al. (2005)
	Heavy AE	+18 mmHg	+12 mmHg^c^ from 30–64 years	Keith et al. (2013)
	Light AE	+14 mmHg		Franklin et al. (1997)
Aortic stiffness (cfPWV)	Light AE	+1.1–1.5 m/s	+1.1–2.0 m/s per +10 years from 40–70 years	Keith et al. (2013)
				Reference Values for Arterial Stiffness’ Collaboration (2010)
Cortisol	Vigorous AE	+70–300% peakΔ	+20–50%	Kanaley et al. (2001)
				Van Cauter et al. (1996)
Inflammation (IL-6)	Vigorous AE	+0.20 pg/ml	+0.16 pg/ml per +10 years from 45–64 years	Tsao et al. (2021)
				Hager et al. (1994)
Strength	Peak torque^d^	−15–20%	−10–15% every 10 years from 45–84 years	Hilbert et al. (2003)
				Ferrucci et al. (2012)

MCA PI, middle cerebral artery pulsatility index; cfPWV, carotid-femoral pulse wave velocity; IL, interleukin.

a secondary regression analysis calculated from Lefferts et al., 2020 data.

b for adults with systolic blood pressure between 120–139 mmHg.

c for adults with systolic blood pressure >160 mmHg.

d peak torque achieved following muscle damaging leg exercise. Data approximated from the following references (Hager et al., 1994; Van Cauter et al., 1996; Franklin et al., 1997; Kanaley et al., 2001; Hilbert et al., 2003; Ogoh et al., 2005; Reference Values for Arterial Stiffness’ Collaboration, 2010 (Boutouyrie, corresponding author); Ferrucci et al., 2012; Keith et al., 2013; Alwatban et al., 2020; Lefferts et al., 2020; Tsao et al., 2021).

In this paper we presented an amalgam of acute exercise literature, including aerobic and resistance exercise across a spectrum of exercise intensities. It is currently unclear whether one specific type of exercise is a better “aging-mimetic” and thus more protective against age-related disease. This gap in understanding reflects methodological limitations [challenges of assessing outcomes during discontinuous exercise (resistance/high-intensity exercise)], and greater attention paid to continuous aerobic over discontinuous aerobic/resistance exercise in the literature. We posit that the exact exercise type is less important than the response it elicits since 1) epidemiological evidence indicates both aerobic and resistance exercise are associated with reduced disease risk (Ross et al., 2016; Bennie et al., 2020) and 2) all forms of exercise disrupt homeostasis (e.g., running, resistance, and high-intensity interval exercise can induce inflammation, increase blood pressure, increase artery stiffness, load bones, damage muscles etc.), and thus may contribute to beneficial adaptations that attenuate physiological aging and reduce disease risk.

The acute effects of exercise are highly variable and may depend, in part, on age. Data indicate that the given response to acute exercise may be preserved (Hogan et al., 2013; Lavin et al., 2020a, 2020b; Luttrell et al., 2020; Rosenberg et al., 2020; MacNeil et al., 2021), exaggerated (Fleg et al., 1985; Fragala et al., 2019; Rosenberg et al., 2020), or blunted (Nordin et al., 2014; Jakovljevic, 2018; Fragala et al., 2019; Rosenberg et al., 2020) with aging, and that these conflicting responses could occur simultaneously depending on the physiological systems in question. This variable effects of age on acute exercise responses may alter the physiological stimulus that elicits adaptations to repeated exercise in older adults. It is possible that either 1) the attenuated physiological response to exercise (e.g., blunted stimulus), or 2) reduced plasticity/sensitivity (Slivka et al., 2008; Greig et al., 2011; Haran et al., 2012) to a similar or exaggerated exercise response could render exercise somewhat less potent or less beneficial among older adults. Indeed, it appears that greater exercise stimuli is required to elicit measurable physiological adaptations among older adults (Fujimoto et al., 2010). Despite reductions in plasticity and altered exercise responses among older adults, exercise training can elicit physiological adaptations in aged individuals (improved muscular, metabolic, cardiovascular function) (Fujimoto et al., 2010; Carrick-Ranson et al., 2014; Vigorito and Giallauria, 2014; Fragala et al., 2019; Green et al., 2021; Grevendonk et al., 2021) that may even be similar to benefits in young adults (Stratton et al., 1994) and ultimately increase cardiorespiratory fitness (Stratton et al., 1994; Woo et al., 2006; Fujimoto et al., 2010). Taken together, data are clear that despite potentially different acute responses and degree of adaptation to exercise, the cumulative effects of exercise are beneficial in older adults and contribute to reduced disease/mortality risk (Bijnen et al., 1998; Sundquist et al., 2004; Carrick-Ranson et al., 2014; Osawa et al., 2021). It should be underscored that the benefits of exercise are wrought over a lifetime of repeated exposure and thus engaging in regular exercise throughout life elicits greater physiological adaptations and health benefits than exercise initiated only later in life (Russ and Kent-Braun, 2004; Fujimoto et al., 2010; Seals et al., 2019; Lavin et al., 2020a).

## Future Directions, Applications, and Conclusion

Future work should seek to leverage technological advances and innovative methods to further explore acute cellular/physiological responses during exercise. The following recommendations are suggested to fill knowledge gaps surrounding the idea of exercise as an aging mimetic and the protective effects of exercise on age-related chronic disease risk: 1) examine mechanisms behind the beneficial effects of low-intensity exercise on age-related chronic disease, which remains under explored owing to more optimal signal-to-noise ratio observed with moderate-to-vigorous intensity exercise; 2) better identify and understand the phenotypic “signature” and physiological disruption caused by discontinuous exercise types (e.g., resistance, high-intensity interval) compared to continuous aerobic exercise; and 3) better understand the role of individual characteristics (age, sex, health status) in governing acute exercise responses and subsequent exercise-induced adaptation. Additionally, research often interrogates acute exercise to gain insight into training-induced adaptations under the guise that responses following acute exercise should be positive and contribute to beneficial long-term adaptations (Dawson et al., 2018; Voss et al., 2020). In reality, it is important to recognize that exercise is a potent disruption of homeostasis that mimics responses seen with aging and age-related chronic disease (i.e., exercise is disruptive and not necessarily immediately beneficial for physiological systems). It is this insult to homeostasis that primes adaptations to protect against chronic, age-related changes and reduce disease risk *over time* (Figure 2D). If research shifts to focus on the homeostatic disruption incurred *during* exercise, we may better understand the stimulus for adaptation and thus the mechanisms that govern adaptations to exercise and prevent age-related chronic disease.

Ultimately, we posit that regular exercise protects against aging and age-related chronic disease because each bout of exercise is, at its essence, an aging mimetic. The resilience and plasticity of the human body permit adaptations to these repeated exercise-induced “aging” stimuli and ultimately prepares the body’s defenses against the stress of aging and age-related chronic disease.

## Data Availability

The original contributions presented in the study are included in the article/Supplementary Material, further inquiries can be directed to the corresponding author.
